# Sampling technique to pool genetic materials of microorganism communities in blue-swimming crab processing plant industry

**DOI:** 10.1016/j.mex.2023.102503

**Published:** 2023-11-29

**Authors:** Asadatun Abdullah, Tati Nurhayati, Fifi Gus Dwiyanti, Sabila Diana Ahmad Sauqi

**Affiliations:** a Department of Aquatic Product Technology, Faculty of Fisheries and Marine Sciences, IPB University, Bogor, Indonesia; b Department of Silviculture, Faculty of Forestry and Environment, IPB University, Bogor, Indonesia; c Department of Food Science and Technology, Faculty of Agricultural Engineering and Technology, IPB University, Bogor, Indonesia

**Keywords:** Easy and rapid genetic material sample-pooling technique for the determination of bacterial communities in the blue-swimming crab processing plant industry, Seafood-processing, Microorganism, Metagenomics, DNA, 16S rRNA, 18S rRNA

## Abstract

The crab and seafood processing industry must fulfill standard requirements for sanitation, hygiene, and good manufacturing methods to ensure the safety of the products and free from foodborne bacteria. However, equipment and processing unit surfaces are challenging to clean optimally, which can cause persistent bacteria to emerge. Eliminating persistent bacteria is the latest challenge in the fish processing industry for optimal disinfection, preventing cross-contamination, and controlling foodborne outbreaks. Microbiological testing in industry has been limited to selective culture-media techniques; thus, a rapid, sensitive, accurate, and routine applicable analytical method is urgently needed. The significant reduction in the costs of high-throughput sequencing technologies supports the possibility of routine applications in the industry. This study aimed to determine the profile of the microbial community on the surface of the production room and blue-swimming crab processing unit equipment using short-read metagenomic techniques. The analysis included the stages of sampling, bacterial incubation, bacterial DNA isolation, sequencing, and bioinformatics analysis. The first important step to increase the possibility of routine adoption in the seafood industry is to reduce the cost, complexity, and time required to complete the analysis. Therefore, in this protocol, we generate a scalable, flexible, cost-effective, and auditable workflow.

• Collection of bacterial samples by swabbing the surface of the equipment using a sterile cotton swab and sterile cloth, which is easy to apply and follow in the blue-swimming crab processing plant industry.
• Effective and efficient sample-pooling is an important step in identifying bacterial communities by metagenomic analysis.

## Graphical abstract

**Figure:**
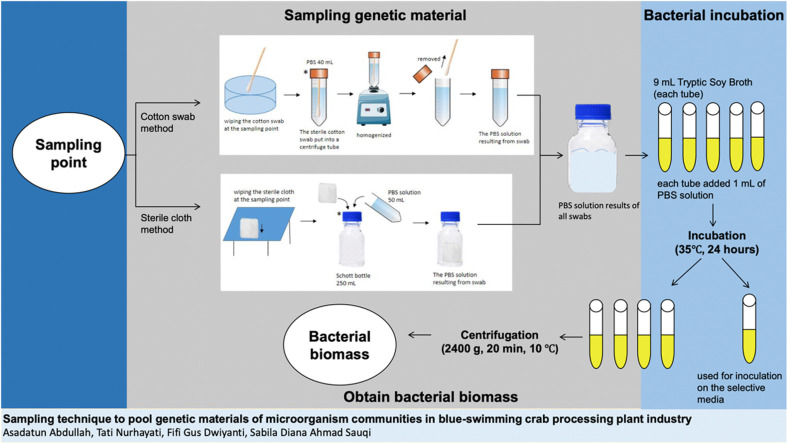

Specifications table

**Table:** 

Subject area:	Biochemistry, Genetics and Molecular Biology
More specific subject area:	Molecular biology
Name of your method:	Easy and rapid genetic material sample-pooling technique for the determination of bacterial communities in the blue-swimming crab processing plant industry
Name and reference of original method:	NA
Resource availability:	All equipment required are described in the method details section

**Method details**

The bacterial communities in the seafood-based production environments influence food quality and safety. It is mandatory to prevent pathogenic and spoilage bacteria in the production room, and must be eliminated by sanitary procedures [1,2]. Bacterial community detection is carried out to determine persistent bacteria in the production environment after sanitation and during the production process [2]. Using metagenomic analysis, identifying microbial communities can detect all bacteria and fungi with low viable cell numbers. The procedure for determining bacterial communities using metagenomic analysis includes the stages of sampling, bacterial incubation, bacterial DNA isolation, sequencing, and bioinformatics analysis [1].

## Materials

Sterile cotton swabs (ONEMED, Indonesia)

Sterile cloth (ONEMED, Indonesia)

Phosphate Buffered Saline (PBS) pH 7.3 (OXOID, England)

Centrifuge tubes 50 mL

Schott bottle 250 mL

Tweezers

Plastic petri dish

Tryptic Soy Broth (TSB)

Vortex

Micropipette (1000 µL)

Filter pipette tips (1000 µL)

Microcentrifuge tubes (1.5 mL)

Clinical centrifuge DLAB DM-0412 (DLAB Scientific Inc., USA)

Laboratory FRIOCELL® Incubators (Medcenter, Munchen)

## Procedure

### Preparation of sampling tools and media

All equipment used in the sampling process must be in sterile condition. Cotton swabs and cloth are in sterile condition provided in the manufacture packaging. The plastic petri dish and the filter pipettes are in sterile condition. Microcentrifuge tubes, tweezers, Schott bottles, and PBS media were sterilized using an autoclave at 121℃ for 15 min. PBS media is made first by dissolving 1 PBS tablet in every 100 mL of distilled water. PBS media (40 mL) was put into each 50 mL centrifuge tube and then sterilized. Sterile equipment and media are ready to be used in the sampling process.

### Determination of sampling points in the production environment and sampling method

**Fig. 1:**
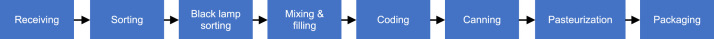
Schematic diagram of the blue-swimming crab meat pasteurized industrial processing plant.

The sampling point is determined before sampling by wiping the surface of the equipment.Determination of sampling points based on a production flow scheme made in the form of a schematic diagram. A schematic diagram of canned crab production can be seen in Fig. 1. Each sampling point has a different sampling method for surface wiping, some use sterile cotton swabs and sterile cloth. The equipment used in each production room is selected as a sampling point, and their sampling method can be seen in Table 1.

**Table 1:** Overview of the different sampling points, sampling methods, and approximate sampling area.

Sampling point	Sampling method	Sampling area
Raw material receiving area		
Table	Sterile cloth	16 × 40 cm
Jar	Cotton swab (J)	3 × 15 cm
Basket	Sterile cloth	16 × 40 cm

Sorting room		
Table	Sterile cloth	16 × 40 cm
Jar	Cotton swab (J)	3 × 15 cm
Processing knives	Cotton swab (M)	2 × 5 cm
Tweezers	Cotton swab (M)	2 × 5 cm
Processing trays	Cotton swab (J)	3 × 15 cm

Black lamp sorting room		
Table	Sterile cloth	16 × 40 cm
Jar	Cotton swab (M)	3 × 15 cm
Tweezers	Cotton swab (M)	2 × 5 cm
Tray	Cotton swab (J)	3 × 15 cm

Canning room		
Table	Sterile cloth	16 × 30 cm
Tray	Cotton swab (J)	3 × 15 cm
Double-Seaming machine	Cotton swab (M)	16 × 40 cm

Coding room		
Coding machine	Sterile cloth	16 × 40 cm
Empty can	Cotton swab (M)	3 × 15 cm

Pasteurization room		
Pasteurization tank	Sterile cloth	16 × 40 cm
Cooler tank	Sterile cloth	16 × 40 cm

Packaging room		
Table	Sterile cloth	16 × 40 cm
Crab's canned product	Cotton swab (M)	3 × 15 cm

Employee equipment		
Hand-gloves	Cotton swab (J)	2 × 5 cm
Apron	Cotton swab (J)	3 × 15 cm

Final canned crab meat		
red meat	Cotton swab (M)	2 × 2 cm
white meat	Cotton swab (M)	2 × 2 cm

*M* = medium, *J* = jumbo.

### Sampling procedure

Sampling was conducted at the canned crab processing plant in Indonesia. Sampling is carried out in two different conditions: when the room is sanitized before production and during the production process. Sampling is carried out by wiping the surface of the specified sampling point using a sterile cotton swab or sterile cloth. The choice of swab tool used is adjusted to the size and area of each sampling point. Sterile cotton swabs are used to wipe manageable equipment with areas that are difficult to swab. Sterile cotton swabs come in two sizes: medium (M) and jumbo (J). The choice of cotton swab size is also adjusted to the size/area of the equipment (sampling point). Another swab tool is a sterile cloth, which is intended for wiping equipment with a wide area, such as sorting tables. How to wipe using each swab tool is described and illustrated as follows:

1. Sterile cloth method
  Sterile cloth is taken from a plastic package in a dry condition. Before use, the sterile cloth is first moistened with PBS solution. Sterile cloth is placed on a plastic petri dish using tweezers and then poured with enough PBS to wet the sterile cloth. Sterile cloth is then placed on the surface of the sample point, the sterile cloth is opened to a width of 16 × 16 cm (maximum area), and wiping is carried out slowly with a sampling area of 16 × 40 cm. The wiping process is carried out using tweezers to avoid direct contact between the hands and the sterile cloth. After swabbing the sampling area, all sterile cloth was put into a 250 mL Schott bottle. All sterile cloth from the swab sampling point was put into the same Schott bottle. 50 mL of PBS solution was added and homogenized by shaking the bottle manually. An illustration of sampling using sterile cloth can be seen in Fig. 2.

**Fig. 2:**
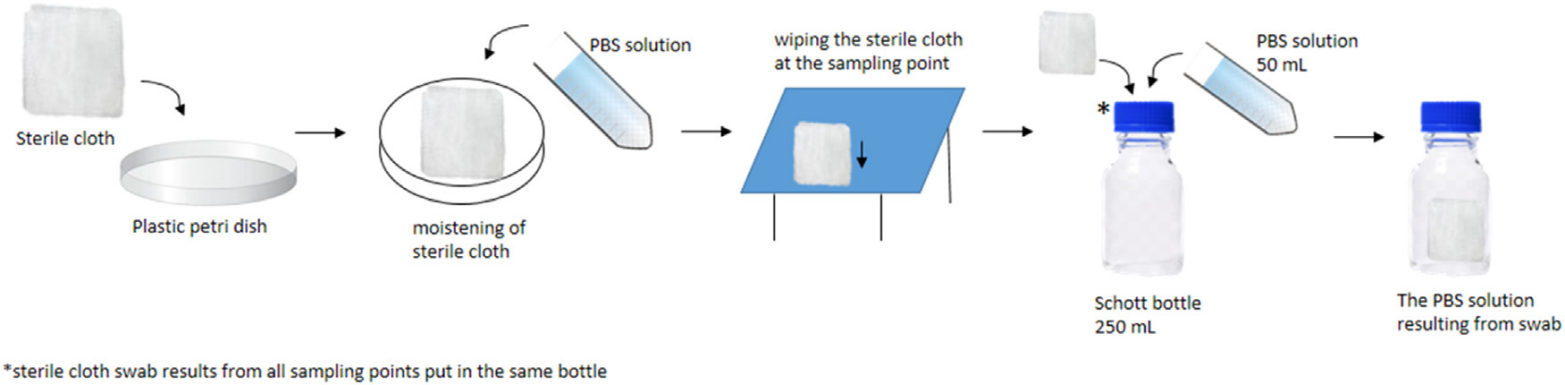
An illustration of sampling using a sterile cloth.
2. Sterile cotton swabs method
  A sterile cotton swab is taken from a plastic package that is still tightly closed. Before being used to wipe the surface of the sample point, a sterile cotton swab is moistened by dipping it in PBS solution. A wet sterile cotton swab is then used by gently wiping the cotton swab at the sampling point according to the sampling area of each sampling point. The sterile cotton swab is then put into a 50 mL centrifuge tube containing 40 mL of PBS solution. One 50 mL centrifuge tube containing 40 mL of PBS solution can be used to store four or five (Medium-sized sterile cotton swab) cotton swab results. The centrifuge tube containing PBS solution and sterile cotton swabs was homogenized using a vortex. The sterile cotton swab was removed from the centrifuge tube, leaving only the PBS solution. An illustration of sampling using a sterile cotton swab can be seen in Fig. 3.

**Fig. 3:**
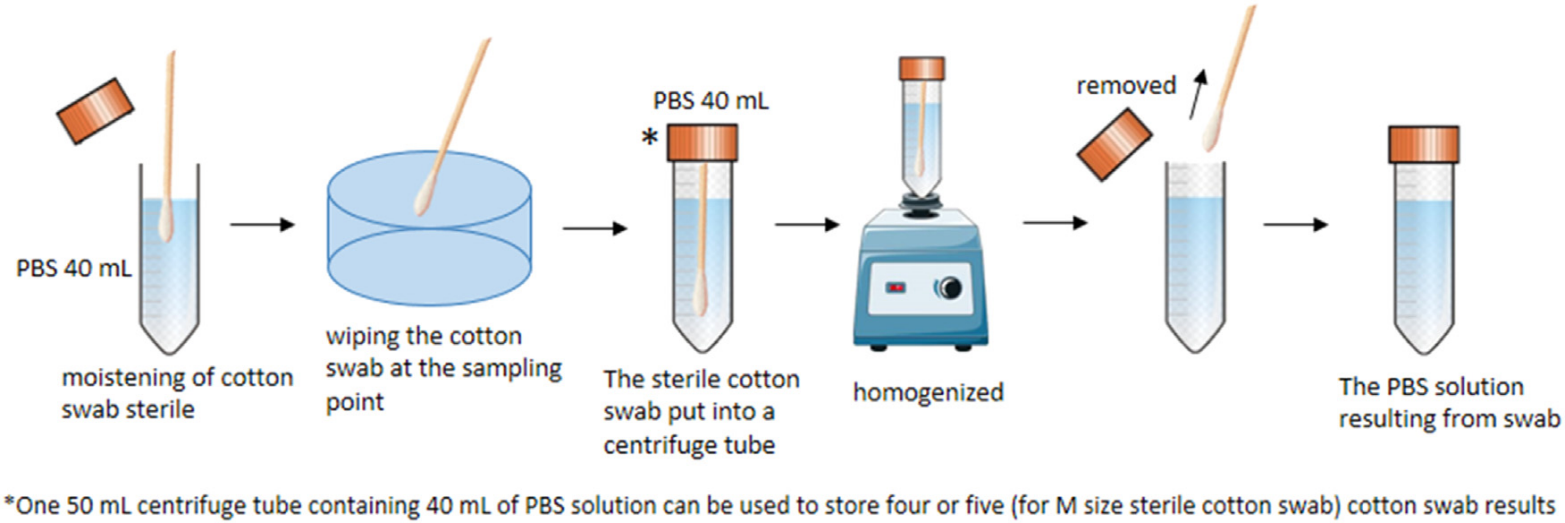
An illustration of sampling using a sterile cotton swab.
  The results of the swab using a cotton swab in a centrifuge tube were combined with the results of the sterile cloth swab into a Schott bottle and then homogenized by turning the bottle upside down. The sterile cloth that is still in the bottle is removed from the Schott bottle. The entire PBS solution from the swab in the tube will be used for inoculation into TSB media. A total of 5 tubes of TSB media with a volume of 9 mL each added 1 mL of PBS solution from the swab using a 1000 µL micropipette. The TSB media was then incubated at 35℃ for 24 h. All of the incubation tubes were centrifuged at 2400 g for 20 min at 10 °C to separate the natant and supernatant. The supernatant liquid is discarded, and pellets are obtained as bacterial biomass, then used for the DNA isolation stage. The bacterial pellet was transferred into a 1.5 mL microcentrifuge tube using a micropipette. Another TSB tube was used for inoculation on the selective media. An illustration of combining PBS solution resulting from a swab using a cotton swab and sterile cloth can be seen in Fig. 4.

**Fig. 4:**
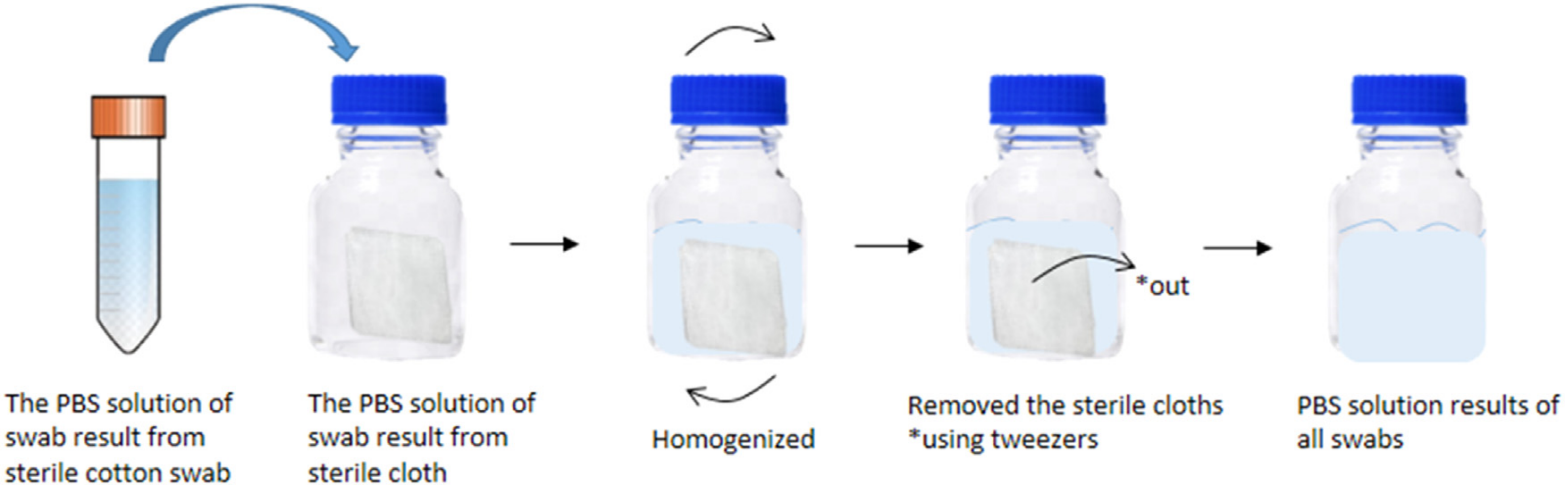
An illustration of combining PBS solution from swabbing using a sterile cotton swab and sterile cloth.

## Validation method

**Fig. 5:**
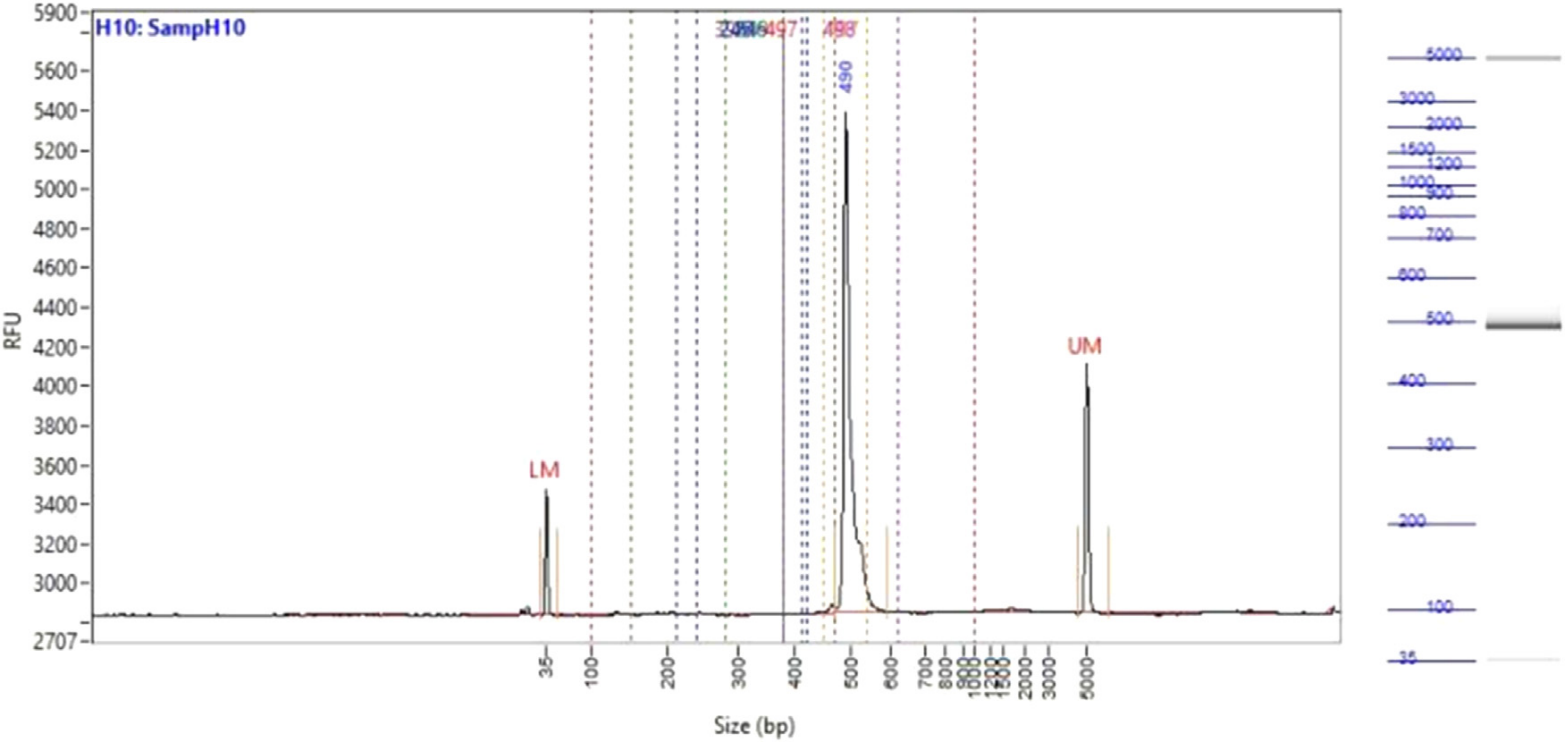
16S rRNA amplicon from bacterial DNA based on our sample-pooling method.

**Fig. 6:**
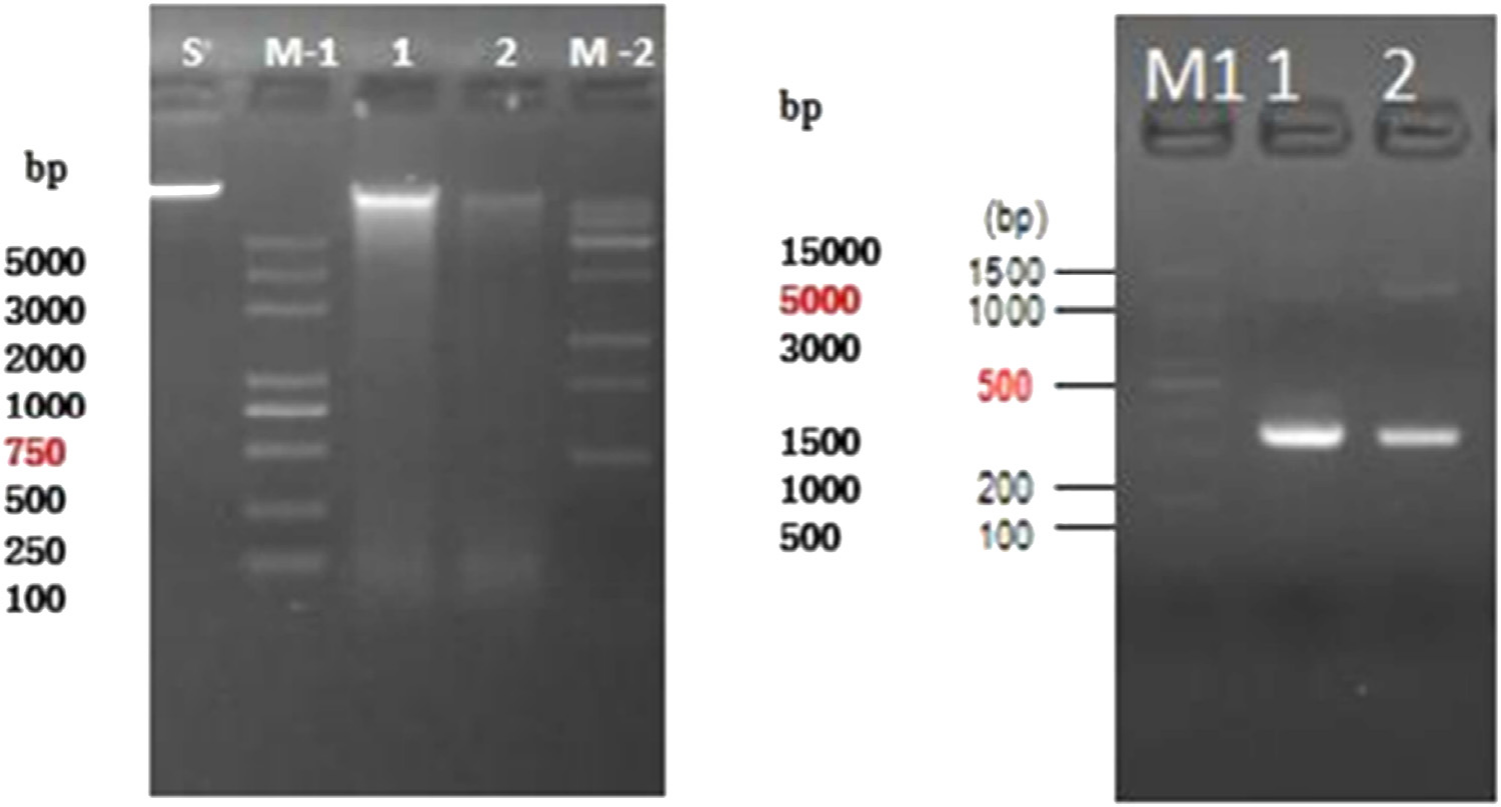
Eukaryote/fungi DNA quality and 18S rRNA amplicon based on sample-pooling method. *S* = Standard sample (50 ng); M-1/M1/M-2= DNA marker; 1 = DNA sample-pool 1; 2 = DNA sample-pool 2.

A rapid and easy method to obtain high quality and quantity of genetic sample-pooling from the blue-swimming crab industry developed in this study (Fig. 1, Fig. 2, Fig. 3, Fig. 4). To exemplify the method above, we performed DNA extraction and amplification of selected gene markers. Genomic DNA from bacteria were extracted using the Presto™ Mini gDNA Bacteria kit (Geneaid Biotech LTD, Taiwan) and genomic DNA from eukaryote/fungi sample were extracted using DNeasy Blood and Tissue Kits (Qiagen, Netherland). The resulting DNA materials were analyzed by using Agarose Gel Electrophoresis and/or the 5400 Fragment Analyzer system (Agilent, United States). The bacterial DNA from the method above was amplified using 16S rRNA V3-V4 regions before metagenomic analysis (PCR results, Fig. 5). Furthermore, eukaryote/fungal DNA from this study method was tested for amplicon sequencing using 18S rRNA V4 (Fig. 6).

## Funding

This study was supported by the 10.13039/501100013877Indonesian Ministry of Education, Culture, Research, and Technology (KEMENDIKBUDRISTEK) with contract ID 102/E5/PG.02.00.PL/2023 to the corresponding author.

## CRediT authorship contribution statement

**Asadatun Abdullah:** Conceptualization, Methodology, Supervision, Writing – review & editing. **Tati Nurhayati:** Investigation, Writing – review & editing. **Fifi Gus Dwiyanti:** Validation, Writing – review & editing. **Sabila Diana Ahmad Sauqi:** Investigation, Data curation, Writing – review & editing.

## Data Availability

Data will be made available on request.
